# COVID-19 and Perioperative Management Strategies for Gastrointestinal Surgery: An Experience From Jiaxing, China

**DOI:** 10.1177/0003134821995087

**Published:** 2022-06

**Authors:** Yuan Zhou, Lusha Cen

**Affiliations:** 1 Department of Gastrointestinal Surgery, 417382 The First Hospital of Jiaxing, The Affiliated Hospital of Jiaxing University, Jiaxing, China; 2 Department of Ophthalmology, The First Affiliated Hospital of Zhejiang Chinese Medical University, Hang Zhou, China

**Keywords:** COVID-19, perioperative management, gastrointestinal surgery

## Abstract

**Background:**

The coronavirus disease (COVID-19) was leading to a worldwide pandemic, which affected surgical operation. This study assessed the efficacy of perioperative management of patients scheduled for gastrointestinal surgery during COVID-19 pandemic of 2020.

**Methods:**

We retrospectively analyzed 188 patients who underwent gastrointestinal surgery during the COVID-19 outbreak in Jiaxing, China. Perioperative data were collected, including data on pre-, intra-, and postoperative management strategies. The same data over the same period in 2019 were also collected for comparison.

**Results:**

A total of 117, 63, and 8 patients underwent emergency, semi-elective, and elective surgeries, respectively. The locals: nonlocals ratio was significantly higher during this investigation period in 2020 than during the same period in 2019 (*P* < .05). After screening, 12 patients were identified as unqualified. The number of gastrointestinal surgeries was reduced in 2020. There were no differences in the ratio of emergency surgery or semi-elective surgery between in 2020 and in 2019. The elective surgery ratio between January 27 and February 28 was found to be lower in 2020 than in 2019 (*P* < .05). The disease spectra of emergency surgery and semi-elective surgery were similar. A total of 31 elective surgeries were postponed. There were five cases of short-term complications for emergency surgeries and two cases of short-term complications for semi-elective surgeries. No long-term complications or COVID-19 infection occurred in any of the cases, and no medical staff member was infected.

**Conclusion:**

Perioperative management strategies minimize the risk of nosocomial infection and reduce the influence of epidemics on gastrointestinal surgery.

Coronavirus disease 2019 (COVID-19) originated in Wuhan, China, in December 2019 and quickly spread to other cities in China and several countries worldwide, leading to a global pandemic.^1^ Coronavirus disease 2019 was subsequently classified as a category B infectious disease, with category A control by the National Health Commission of China, on January 20, 2020, which means that the management of COVID-19 is strictly regulated by law. Many cities worldwide have also implemented rigorous control measures to prevent further spread of the disease. As Jiaxing has a well-developed transportation infrastructure and is located within 100 km from cities such as Shanghai (east), Suzhou (north), Huzhou (west), and Hangzhou (south), the risk of COVID-19 transmission is high. The first case of COVID-19 was confirmed in Jiaxing on January 27, 2020. As of April 1, 2020, a total of 50 cases, including 4 overseas-imported cases, have been confirmed. The First Hospital of Jiaxing, designated as the sole treatment unit for COVID-19 patients in the region, undertakes the diagnosis and treatment of all COVID-19 patients. As such, it faces a higher risk of nosocomial infection.

According to recent research, the main routes of COVID-19 transmission are via respiratory droplets and close contact.^2^ Furthermore, several studies have shown that COVID-19 could be detected in the feces of infected patients,^3,4^ indicating a possibility of fecal-oral transmission. As a special isolation room is often not present in a general surgical ward, appropriate perioperative management strategies for gastrointestinal surgery during the COVID-19 outbreak period are crucial in minimizing nosocomial infection risk and reducing the influence of the pandemic on gastrointestinal surgery.

In this study, we aimed to analyze the perioperative management strategies undertaken for patients who underwent surgery for gastrointestinal diseases in The First Hospital of Jiaxing during the COVID-19 outbreak. It is anticipated that our findings will help other surgeons with the perioperative management of their patients during pandemics.

## Patients and Methods

### Patients

This retrospective clinical study was conducted on 188 patients who underwent gastrointestinal surgery during the COVID-19 outbreak in Jiaxing, China, between January 1, 2020 and March 30, 2020. Clinical data on the gastrointestinal surgeries performed between January 27, 2019 and March 30, 2019 were collected. Informed consent was obtained from all participants, and the study was approved by the Ethics Committee of The First Hospital of Jiaxing.

The inclusion criteria were as follows: (1) emergency surgery for patients with clear surgical indications of acute abdomen, (2) semi-elective surgery for patients with malignant tumors or patients requiring surgical treatment within a specified time limit, and (3) elective surgery for patients who insisted on surgery despite the doctor’s recommendation for postponement until the outbreak situation improves. The exclusion criteria were as follows: (1) surgical contraindications, including acute myocardial infarction, blood coagulation disorders, and cardiopulmonary insufficiency; and (2) refusal of surgery.

### Pre-Operation Management

The preadmission screening, performed by trained medical professionals, included: (1) epidemiological investigations, including a contact history with COVID-19 patients or travel history to epidemic areas; (2) clinical signs and symptoms^5,6^ such as fever, cough, and other indications of a respiratory tract infection; and (3) body temperature measurement and chest computed tomography (CT) scan. The CT report was reviewed by 2 trained radiologists. Any abnormality found in the preadmission screening was assessed by an expert COVID-19 group formed for the sampling, diagnosis, and treatment of COVID-19 patients. This would help us decide on the appropriate measures to be taken for further treatment. Other preoperative assessment was conducted according to ASA physical status classifications. Cardiac and pneumonic functions were evaluated by electrocardiogram. Laboratory studies were include a complete blood count and renal and liver function tests.

**Figure 1.**
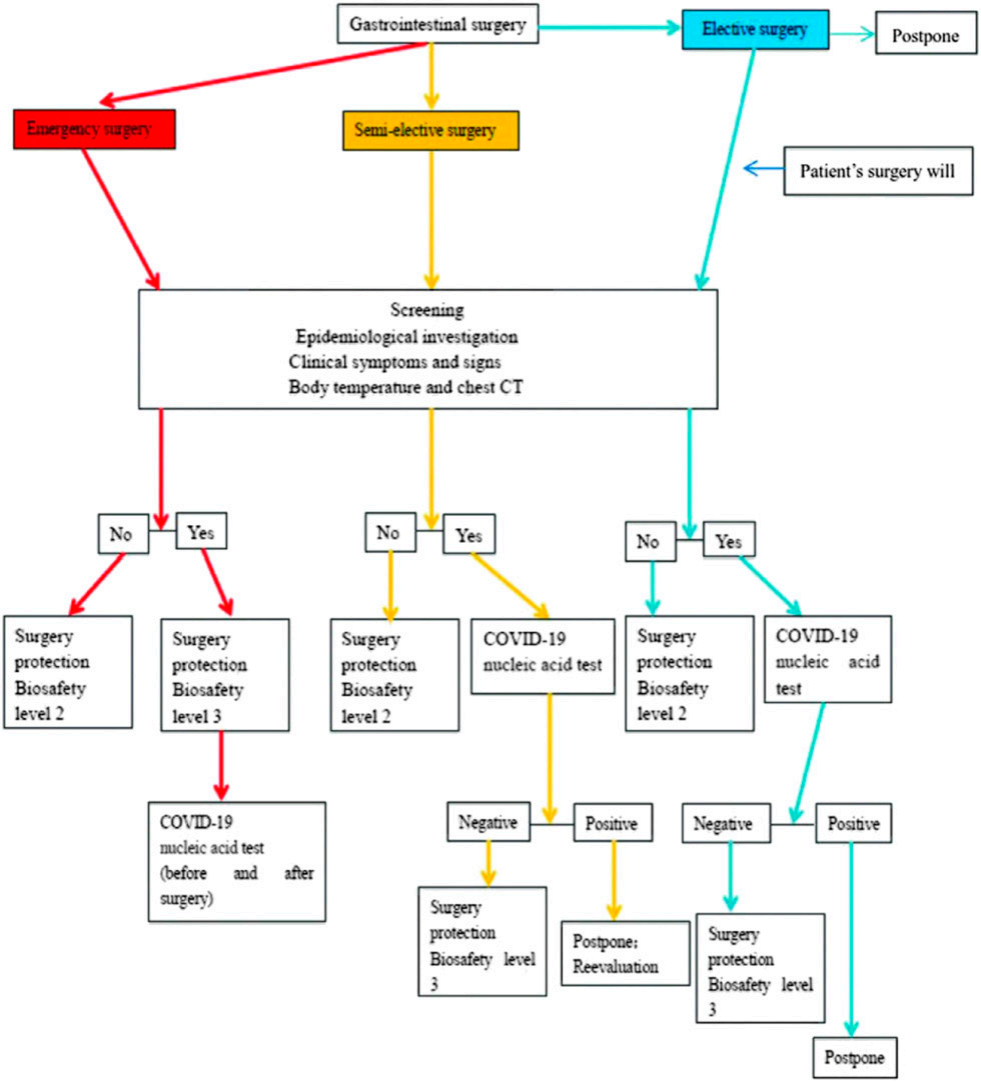
Flowchart depicting the preoperation management of patients.

The surgical grading management, if patients were not suspected of COVID-19 infection, was as follows: (1) emergency surgery performed with secondary protection, as was done previously; (2) semi-elective surgery performed with secondary protection; or (3) elective surgery postponed until the outbreak situation eases, or performed with secondary protection if patients insisted on surgery. Even if patients were suspected of COVID-19 infection, emergency surgery was performed without waiting for the nucleic acid test results, but with tertiary protection. The patient was temporarily isolated in a special surgical ward before the test result was available. A nucleic acid test was required before and after surgery. If any of the two results was positive, the patient was isolated in a special negative pressure ward for the simultaneous treatment of COVID-19 infection. If the result was negative, the patient was isolated in the special surgical ward, and the nucleic acid test was repeated on the basis of the status of the illness. For patients suspected of having COVID-19 infection, semi-elective surgery was conducted after the nucleic acid test results were obtained. If the test result was negative, surgery was carried out with tertiary protection. After surgery, the patient was isolated in the special surgical ward, and the nucleic acid test was repeated. Otherwise, the operation was postponed, and the patient was transferred to the infectious diseases department for the treatment of COVID-19 infection. Elective surgery was postponed for patients suspected of having COVID-19 infection. The test sample was collected using a nasopharyngeal test paper. The test was performed using a novel coronavirus nucleic acid detection kit (Hunan Shengxiang Biotechnology Co. Ltd), and the results were obtained in 6 hours (Figure 1)*.*

### Intraoperative Protective Measures

Evaluation by surgical nurse: Epidemiological history and measurement of body temperature were included in the preoperative evaluation by a surgical nurse. The indications for operation were reevaluated if the information was not consistent with that in previous records. Patients suspected of or with confirmed COVID-19 infection were accommodated in a negative pressure operating room.

Secondary protection is the conventional approach including use of disposable hats, disposable isolation clothing, and sterile gloves. Tertiary is an additional layer of protection by using double disposable hats, disposable isolation clothing or medical protective clothing, shoe cover (long style), N95 mask, goggles or face screen protection, and sterile double gloves.

Others: The number of surgical assistants was kept to the minimum according to surgery type. Visitors were not allowed to enter the operating room. The sampling frequency of the air and inspection frequency of the surgical equipment surfaces were increased.

### Postoperative Management

Postoperative handling of the patient, including dressing changes and drainage tube and suture removal, was carried out with secondary protection. Differential diagnoses of non–surgery- or surgery-related fever were needed. The expert COVID-19 group was consulted if a suspicious COVID-19 case was encountered.

### Ward Control Measures

The number of patients in each ward was kept to the minimum. A single ward was arranged whenever possible. Special isolation wards were prepared for the temporary placement of patients suspected of COVID-19 infection. Patients with confirmed COVID-19 infection were placed in a special isolation unit managed by specialists. Access to the ward was strictly controlled, and no visitors were allowed. Patients and caregivers were required to wear masks. A daily temperature check for caregivers was also implemented, and the disinfection scope and frequency were increased.

### Postoperative Follow-Up

Postoperative follow-up was carried out through teleconferencing and telephone calls to identify any COVID-19-related symptoms.

### Statistical Analysis

Statistical analysis was carried out using SPSS (version 17.0) software. Pearson’s chi-square test was performed, and *P* values <.05 were considered statistically significant.

## Results

### Patient Characteristics

A total of 121 men and 67 women, with an average age of 55.4 years (range: 5-89 years), were included in the present study. There were 117 cases of emergency surgery, 63 cases of semi-elective surgery, and 8 cases of elective surgery. Among the patients, 166 were local residents and 22 were nonlocals (Table 1). The locals: nonlocals ratio was significantly higher during this investigation period in 2020 than during the same period in 2019 (*P* < .05), especially during the period between January 27 and February 28 (*P* < .05).

**Table 1.** Regional Distribution of Patients Between 2019 and 2020 (Locals/Nonlocals).

Period	2019	2020	χ^2^	*P*
January 27-February 28	91/24	65/7	3.979	.046
March 1-March 30	125/33	101/15	2.930	.087
Total	216/57	166/22	6.603	.01

### Screening Results

A total of 12 patients were excluded from the study. Eleven patients in the emergency surgery group were excluded, including 1 patient with a CT scan that showed a “glass-like shadow,” 9 patients with a fever, and 1 patient with a travel history to a COVID-19 epidemic area. One patient in the semi-elective surgery group was excluded owing to fever (Table 2).

**Table 2.** Summary of Screening Results.

Surgery (n)	Unqualified total	Screening Type		
		Computed tomography	Fever	Epidemiology
Emergency surgery(117)	11	1	9	1
Semi-elective surgery(63)	1	0	1	0
Elective surgery(8)	0	0	0	0

### Assessment of the Results and Treatment Measures of the Unqualified Cases by the Expert COVID-19 Group

The 12 patients who were excluded from our study were referred to the COVID-19 expert group for consultation and assessment. The results of the nucleic acid tests were negative, indicating that there were no COVID-19 cases in the emergency surgery group. Assessment by the expert group revealed that the “glass-like shadow” on the CT scan was indicative of interstitial pneumonia. In addition, 8 patients with a fever were found to have intraperitoneal inflammation, and 1 patient with a fever was found to have a general pulmonary infection. The nucleic acid test result of the excluded semi-elective surgery patient was also negative. Assessment by the expert group suggested that the patient had an upper respiratory tract infection. These patients were transferred to and isolated in the special surgical ward for postoperative follow-up treatments following their surgery.

### Surgery Type Comparison Between Outbreak and Remission Period of COVID-19

We defined the period between January 27, 2020 and February 28, 2020 as the outbreak period, and that between March 1, 2020 and March 30, 2020 as the remission period. Compared to the outbreak period, the number of emergency, semi-elective, and elective surgeries increased during the remission period.

#### Distribution of emergency surgery types

During the outbreak period, 46 cases of emergency surgeries were performed, including those for acute appendicitis (n = 13), gastrointestinal perforation (n = 27), and intestinal obstruction (n = 6). During the remission period, the total number of cases was 71 and included acute appendicitis (n = 54), gastrointestinal perforation (n = 8), intestinal obstruction (n = 4), incarcerated hernia (n = 2), and abdominal trauma (n = 3). Incarcerated hernia and abdominal trauma were the newly added disease types during the remission period.

#### Distribution of semi-elective surgery types

During the outbreak period, 26 patients underwent semi-elective surgeries, including those with gastric cancer (n = 5), colorectal cancer (n = 18), and abdominal malignancy (n = 3). During the remission period, the total number of cases was 37, including gastric cancer (n = 8), colorectal cancer (n = 26), abdominal malignancy (n = 2), and intestinal malignant tumor (n = 1).

#### Distribution of elective surgery types

There were no elective surgeries performed during the outbreak period. During the remission period, eight elective surgeries were conducted, including surgeries for a colorectal benign tumor (n = 2), closure of ostomy (n = 4), and rectocele (n = 2).

### Graded Surgery Amount Comparison Between 2019 and 2020

The number of graded surgeries decreased in 2020 compared to that in 2019, during the period between January 27 and February 28 and between March 1 and March 30.

#### The period between January 27 and February 28

There were no significant differences in the ratios of emergency surgery or semi-elective surgery in 2020 compared to those in 2019 during the period between January 27 and February 28. The elective surgery ratio was significantly lower in 2020 than in 2019 (*P* < .05) (Table 3).

Period from March 1 to March 30

**Table 3.** Graded Surgery Comparison Between 2019 and 2020 (January 27 to February 28).

Surgery level	2019	2020	χ^2^	*P*
Emergency surgery	67	46	.587	.444
Semi-elective surgery	29	26	2.531	.112
Elective surgery	19	0	13.241	.000
Total	115	72		

There were no significant differences in the ratios of emergency surgery, semi-elective surgery, or elective surgery in 2020 compared to those in 2019 during the period from March 1 to March 30 (Table 4).

**Table 4.** Graded Surgery Comparison Between 2019 and 2020 (March 1 to March 30).

Surgery level	2019	2020	χ^2^	*P*
Emergency surgery	97	71	.001	.975
Semi-elective surgery	43	37	.709	.400
Elective surgery	18	8	1.574	.210
Total	158	116		

### Surgery Type Comparison Between 2019 and 2020

The disease spectrum of emergency and semi-elective surgeries between January 27 and March 30 was the same in 2019 and 2020.

#### Distribution of emergency surgery types

**Figure 2.**
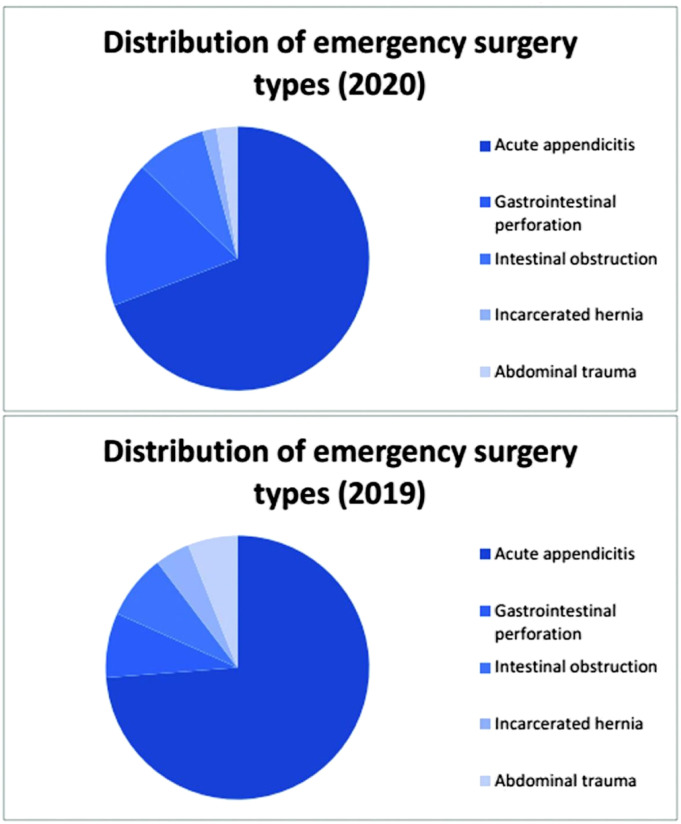
Distribution of emergency surgery types.

There were 117 emergency surgeries for acute appendicitis (n = 81), gastrointestinal perforation (n = 21), intestinal obstruction (n = 10), incarcerated hernia (n = 2), and abdominal trauma (n = 3) in 2020. During the same period in 2019, there were 164 cases of acute appendicitis (n = 121), gastrointestinal perforation (n =13), intestinal obstruction (n = 13), incarcerated hernia (n = 7), and abdominal trauma (10) (Figure 2).

#### Distribution of semi-elective surgery types

**Figure 3.**
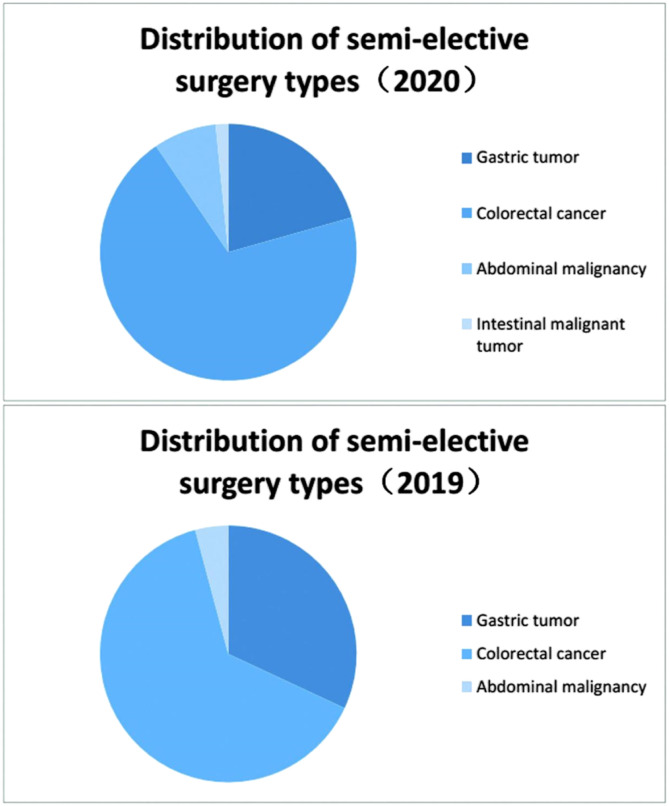
Distribution of semi-elective surgery types.

There were 63 semi-elective surgeries for gastric cancer (n = 13), colorectal cancer (n = 44), abdominal malignancy (n = 5), and intestinal malignant tumor (n = 1) in 2020. During the same period in 2019, there were 72 cases of gastric cancer (n = 23), colorectal cancer (n = 46), and abdominal malignancy (n = 3) (Figure 3).

#### Distribution of elective surgery types

**Figure 4.**
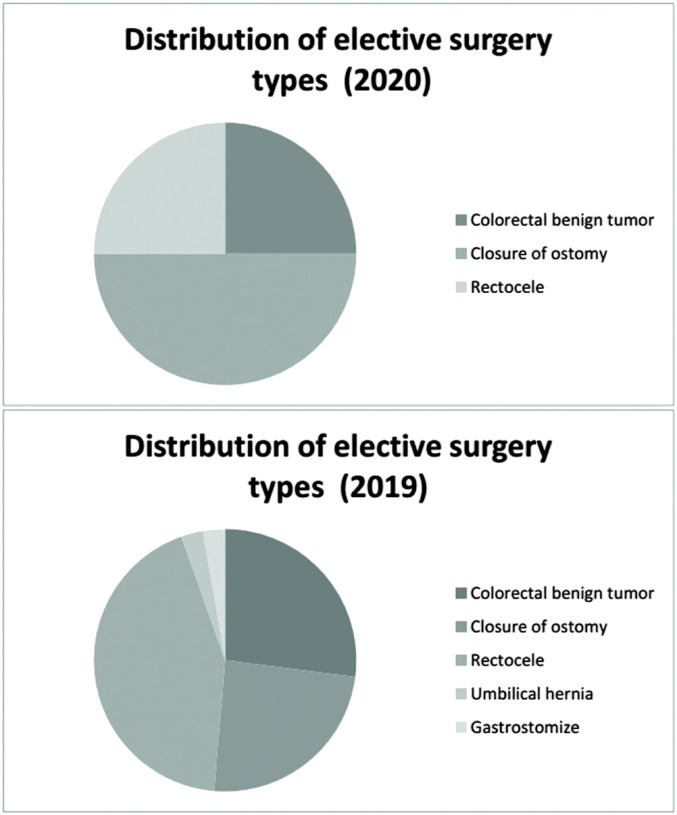
Distribution of elective surgery types.

There were 8 elective surgeries for colorectal benign tumors (n = 2), closure of ostomy (n = 4), and rectocele (n = 2) in 2020. During the same period in 2019, there were 37 cases of colorectal benign tumor (n = 10), closure of ostomy (n = 9), rectocele (n = 16), umbilical hernia (n = 1), and gastrostomies (n = 1) (Figure 4).

### Postponing Surgery Types

Thirty-one elective surgeries were delayed, including those for benign colorectal tumors (n = 14), closure of ostomy (n = 6), rectocele (n = 9), incisional hernia (n = 1), and artificial fistula-induced hernia (n = 1).

### Perioperative Infection and Postoperative Complications

Out of the 117 emergency surgeries, there were 5 cases of short-term complications; 3 of surgical incision infection, 1 of intraperitoneal infection, and 1 of venous thrombosis of the lower limbs. Out of the 63 elective surgeries, there were 2 cases of short-term complications; 1 of intraperitoneal infection and 1 of pulmonary infection. All patients recovered after timely treatment. No long-term complications were found in any of the cases. Additionally, none of the patients and medical staff had COVID-19 infection.

## Discussion

Coronavirus disease 2019 is a life-threatening respiratory disease with a long incubation period and high infectivity. Some patients have been shown to be infected by nosocomial transmission.^7^ The latest research suggested that truly asymptomatic or temporarily asymptomatic (due to virus incubation period) patients are also contagious.^8^ In addition, the diagnostic accuracy may be influenced by false-negative nucleic acid test results^9,10^ and concealment of patient contact history. While it is challenging for gastrointestinal surgeons to work smoothly during the pandemic, we demonstrated that the measures that our surgery team had undertaken minimized the risk of nosocomial infection and reduced the impact of COVID-19 on gastrointestinal surgery.

Preoperation management is the first and most vital step in isolating COVID-19 patients from the surgical ward. Epidemiological history is very important for screening high-risk patients in a non-epidemic area like Jiaxing. For the most part, however, the accuracy of epidemiological history depends on the cooperation level of the patient. A surgical nurse double-checked the patient’s history before sending them to the operation room. A majority of the patients in our study were screened by temperature measurement since it is the most economical and convenient method of screening.^11^ Chest CT examination was also performed routinely as part of our preoperative management. Computed tomography reports provide important evidence that forms the basis for the diagnosis of COVID-19.^12^ Preliminary screening avoids the use of extensive nucleic acid testing which can incur high economic costs. Preadmission screening was carried out for all patients; 12 out of the 188 patients were screened out in the current study.

Furthermore, standardized nucleic acid sampling was used to improve the quality of samples and reduce the false-negative rate.^13^ The enhanced diagnostic accuracy of this method would mitigate the risk of infection among noninfectious diseases specialists such as surgeons. In our study, suspected cases of COVID-19 infection were identified after consultation with the expert group, and these patients were isolated in a special surgical ward. Reducing human-to-human contact has been shown to be an effective means of limiting the transmission of the disease.^14^ Furthermore, we strictly controlled the number of people in the operating room and wards. Our management strategy largely involved screening for suspected cases of COVID-19 infection to minimize the risk of disease transmission. None of the patients in our department were missing diagnosis or misdiagnosis of COVID-19.

As much as possible, we aimed to achieve benefit maximization for the patients and alleviate the influence of the epidemic on gastrointestinal surgery. Through surgical grading management, we differentiated the three levels of surgeries. Although the number of gastrointestinal surgeries was generally reduced, none of the emergency surgeries was delayed. Additionally, the ratio of emergency surgery or semi-elective surgery was unchanged during both the outbreak and remission periods in 2020 compared with the same periods in 2019. Furthermore, no postoperative COVID-19 infection was identified and no long-term complications were found in the patients. Last, no medical staff was infected.

The COVID-19 outbreak coincided with the traditional Chinese New Year holiday. Isolation at home with a more regular diet may have reduced number of gastrointestinal emergencies such as gastrointestinal perforation. Wang et al^15^ conducted an online survey including 1210 respondents from 194 cities in China, showing about one-third reported moderate-to-severe anxiety, and more than half of the respondents rated the psychological impact as moderate to severe during the initial phase of the COVID-19 outbreak in China. Furthermore, anxiety arising from the global pandemic may have also resulted in fewer patients seeking medical attention and treatment, thereby explaining a reduced number of elective surgeries. Most elective surgeries were postponed during the outbreak and remission periods, with none being performed during the outbreak period and 8 being performed during the remission period.

Effective prevention and control require the full attention of the local government, private sector, and general population.^16^ The local government imposed strict control of relevant communities that were affected by COVID-19, including patients and individuals who had close contact with patients or people from a known COVID-19 pandemic area. The limitation of this study is the lack of surgical experience with confirmed cases of COVID-19 infection. We have to stay vigilant and comply strictly with the diagnostic and treatment measures in place. These control measures will ensure the smooth operation as well as the safety of our medical staff and patients during this epidemic. We hope that our experience will help other surgeons with the perioperative management of their patients for gastrointestinal surgery during the COVID-19 outbreak.

## References

[1] Wang C Horby PW Hayden FG, et al. A novel coronavirus outbreak of global health concern. Lancet 2020;395:470-473. 31986257 10.1016/S0140-6736(20)30185-9 PMC7135038

[2] Rothan HA Byrareddy SN. The epidemiology and pathogenesis of coronavirus disease (COVID-19) outbreak. J Autoimmun 2020;109:102433. 32113704 10.1016/j.jaut.2020.102433 PMC7127067

[3] Cholankeril G Podboy A Aivaliotis VI, et al. High prevalence of concurrent gastrointestinal manifestations in patients with SARS-CoV-2: Early experience from California. Gastroenterology 2020;159(2):775-777. S0016-5085:30471-30476. 32283101 10.1053/j.gastro.2020.04.008 PMC7194555

[4] Zhang H Kang ZJ Gong HY, et al. Digestive system is a potential route of COVID-19: An analysis of single-cell coexpression pattern of key proteins in viral entry process. Gut 2020;69:1010-1018.

[5] Huang C Wang Y Li X, et al. Clinical features of patients infected with 2019 novel coronavirus in Wuhan, China. Lancet 2020;395:497-506. 31986264 10.1016/S0140-6736(20)30183-5 PMC7159299

[6] Jiang SB Xia S Ying TL, et al. A novel coronavirus (2019-nCoV) causing pneumonia-associated respiratory syndrome. Cell Mol Immunol 2020;17:554. 32024976 10.1038/s41423-020-0372-4 PMC7091741

[7] Wang DW Hu B Hu C, et al. Clinical characteristics of 138 hospitalized patients with 2019 novel coronavirus-infected pneumonia in Wuhan, China. JAMA 2020;323:1061-1069. 32031570 10.1001/jama.2020.1585 PMC7042881

[8] Rothe C Schunk M Sothmann P, et al. Transmission of 2019-nCoV infection from an asymptomatic contact in Germany. N Engl J Med 2020;382: 970-971. 32003551 10.1056/NEJMc2001468 PMC7120970

[9] Zhang R Li JM. The way to reduce the false negative results of 2019 novel coronavirus nucleic acid detection. Zhong hua Yi Xue Za Zhi 2020;100:801-804. 10.3760/cma.j.cn112137-20200215-00288 32234149

[10] Wu J Liu J Li S, et al. Detection and analysis of nucleic acid in various biological samples of COVID-19 patients. Travel Med Infect Dis 2020;18:101673. 10.1016/j.tmaid.2020.101673 PMC7165102 32311437

[11] Mogensen CB Vilhelmsen MB Jepsen J, et al. Ear measurement of temperature is only useful for screening for fever in an adult emergency department. BMC Emerg Med 2018;18:51. 30509206 10.1186/s12873-018-0202-5 PMC6276133

[12] Li Y Xia L. Coronavirus disease 2019 (COVID-19): Role of chest CT in diagnosis and management. AJR Am J Roentgenol 2020;214:1280-1286. 32130038 10.2214/AJR.20.22954

[13] Ye B Fan C Pan Y, et al. Which sampling method for the upper respiratory tract specimen should be taken to diagnose patient with COVID-19? Zhonghua Er Bi Yan Hou Tou Jing Wai Ke Za Zhi 2020;55(6):583-588. 32166939 10.3760/cma.j.cn115330-20200223-00116

[14] Chakraborty I Maity P. COVID-19 outbreak: Migration, effects on society, global environment and prevention. Sci Total Environ 2020;728:138882. 32335410 10.1016/j.scitotenv.2020.138882 PMC7175860

[15] Wang C Pan R Wan X, et al. Immediate psychological responses and associated factors during the initial stage of the 2019 Coronavirus disease (COVID-19) epidemic among the general population in China. Int J Environ Res Public Health 2020;17(15):1729. 10.3390/ijerph17051729 PMC7084952 32155789

[16] Swerdlow DL Finelli L. Preparation for possible sustained transmission of 2019 novel coronavirus: Lessons from previous epidemics. JAMA 2020;323:1129-1130. 32207807 10.1001/jama.2020.1960

